# Early postoperative mortality in the elderly: a pilot study

**DOI:** 10.1186/1756-0500-2-118

**Published:** 2009-07-01

**Authors:** Irene Asouhidou, Theodora Asteri, Petros Sountoulides, Konstantinos Natsis, George Georgiadis

**Affiliations:** 1 2nd Department of Anesthesia "G. Papanikolaou" Regional Hospital, Thessaloniki, Greece; 2 Department of Cardioanesthesia "G. Papanikolaou" Regional Hospital, Thessaloniki, Greece; 3 Medical School, University of California, Irvine, USA; 4 Department of Anatomy, Medical School of Aristotle University of Thessaloniki, Greece

## Abstract

**Background:**

As the population ages and as surgical and anaesthetic techniques advance, more elderly people will be referred for surgery. Postoperative mortality and morbidity are certainly associated with increasing age; however the severity of coexisting medical conditions is an equally important risk factor. In the present study we tried to look into the aetiology of early postoperative morbidity of elderly patients following major surgery, in relation to their medical history.

**Findings:**

Fifty patients aged 70 to 95 years of age were enrolled in the study. All patients had undergone major elective orthopedic procedures due to either osteoarthritis of femoral head or femoral neck fracture. Patients were followed up by telephone interview one month following their discharge. 8 out of 50 patients (16%) were reported dead at follow up. For the majority of the patients who died, the cause of death was directly related to their previous medical history.

**Conclusion:**

Despite the detailed preoperative evaluation, and the intensive intraoperative and early postoperative anaesthetic care, most patient's deaths were related to their previous medical history.

## Background

It is estimated that by the year 2040, people over the age of 65 will constitute approximately 24% of the earth's population. Half of them will need to undergo certain surgical procedures in view of the fact that perioperative morbidity has a 3-fold increase in this subset of patients [1].

It becomes quite challenging to distinguish between changes in physiology caused by aging, and the pathophysiology of certain diseases that are very common in the elderly. Functional reserve and maximal capacity of all major organ systems are significantly reduced and may not be able to meet the increased demands associated with acute illness surgery [2]. Postoperative mortality is thought to be associated with increasing age; however the severity of coexisting medical problems seems to be an equally important risk factor [3-5]. The purpose of this study is to evaluate the effect of previous medical conditions on the mortality rate of elderly patients during the early postoperative phase following major orthopedic surgery.

## Method

All patients (n = 50) aged 70 to 95 years old, that were admitted to our hospital for osteoarthritis of the femoral head or femoral neck fracture between January 2004 to May 2004, were enrolled in the study. The operations performed were total hip arthroplasty for those suffering from osteoarthritis of femoral head and internal fixation and hemiarthroplasty of the hip for those suffering from transtrochanteric and femoral neck fracture respectively. None of the patients were excluded.

Every patient was preoperatively evaluated and classified according to the physical status classification of the American Society of Anesthesiology (ASA) [5]. The preoperative clinical evaluation, the diagnostic work-up and the management of cardiovascular diseases were performed according to the American College of Cardiology and American Heart Association (ACC/AHA) guidelines [6]. The type of anesthesia that was used was mainly regional. Patient characteristics, perioperative assessments according to the ASA, the type of anesthesia and the type of operations performed were summarized from the patient's clinical charts and are presented in Table 1.

**Table 1:** Demographic data

**Characteristics**	**No**	**%**
Patients	50	100

Age (mean yrs)	79 ± 6	

Male/Female	28/22	56/44

ASA II	16	32

ASA III	17	34

ASA IV	17	34

**Type of Anaesthesia used:**		

General (g)	5	10

Spinal	40	80

Epidural (e)	3	6

Combined: g/e	2	4

		

**Orthopedic lesions:**		

Osteoarthritis	11	22

Femoral neck fracture	17	34

Transtrohaderic	22	44

		

**Orthopedic procedure:**		

total hip replacement	11	

hemiarthroplasty	17	

internal fixation	22	

During surgery, basic intraoperative monitoring (ECG, SpO_2_, EtCO_2_, non invasive blood pressure-NIBP) and invasive blood pressure-IBP was applied to all patients. Central venous catheter or pulmonary catheter was performed selectively in patients with increased risk; i.e preexisting cardiac heart failure or heart valvular disease.

Intraoperative anesthesiological complications (changes in blood pressure, heart rate) were also recorded. Intraoperative, a fall in blood arterial pressure (BAP) of more than 30% was considered clinically significant and was initially managed with infusion of crystalloids (Ringer Lactate and Normal Saline 0,9%) in order to maintain the appropriate intravascular volume and vasoconstriction (phenylephrine). The hemoglobin threshold for blood transfusion was designated to be 8 mg.dL^-1 ^and 10 mg.dL^-1 ^for patients with coronary artery disease.

The mean length of hospitalization was 6–10 days. The status of all the patients was checked by telephone interview one month after their discharge from the hospital. Data regarding the outcome of those patients were collected from their medical hospital charts and death certificates and were analyzed statistically using the x^2^-test. Differences were considered significant at *p *value < 0.05.

The pre-assessment and the intraoperative management of the patients were performed by the first author.

## Results

During the period of 30 days, 8 out of 50 patients (16%) were reported dead on follow up, their mean age being 85 ± 6 years. Mortality at 30 days was 6% (n = 3) in women and 10% (n = 5) in men, with no significant difference (p > 0.5).

One patient (pt I-Table 2) with a history of a myocardial infarction (MI) more than 5 years ago and heart failure with a moderately good ejection fraction (EF:55%), suffered a fatal pulmonary edema. He had not experienced any symptoms of coronary ischemia after MI and he met a 6-MET (metabolic equivalent levels) level before surgery.

**Table 2:** Data of diseased patients (pt).

	**Pt I**	**ptII**	**ptIII**	**ptIV**	**ptV**	**ptVI**	**ptVII**	**ptVIII**
**ASA**	3 MI, HF	2 AY	2 AY	3 Aortic valve stenosis	3 COPD, RHF	3 COPD, hypothyroidism	2 No medical history	2 No medical history

**anesthesia**	spinal	spinal	spinal	general	spinal	spinal	spinal	spinal

**Invasive monitoring**	no	No	no	Swan-Ganz	Central venous	Central venous	no	no

**transfusion**	yes	No	yes	yes	yes	yes	yes	yes

**complication**	hypotension	-	-	hypotension	hypotension	-	hypotension	-

MI: Myocardial infraction, HF: heart failure, AY: arterial hypertension, RHF: right heart failure, COPD: chronic obstructive pulmonary disease.

Acute myocardial infarction and cardiac arrest was the cause of death in two patients (pts II&III-Table 2) with the only incriminating evidence from their history being arterial hypertension.

A female patient (pt IV-Table 2) with stenosis of the aortic valve died of acute MI. Preoperative tests revealed an ejection fraction (EF) of 55%, with normal wide pressure and systolic arterial pressure about 150 mmHg. She had moderate functional capacity and met a 5-MET level during her normal daily activities.

Acute heart failure was the cause of death in a patient with coexisting chronic obstructive pulmonary disease and right heart failure (pt V-Table 2). He had a normal chest X-ray and spirometry revealed decreased Forced Vital Capacity (FVC), decreased Forced End-expiratory Volume at 1 minute (FEV_1_) and decreased Forced Expiratory Flow Rate (FEF_25–75%_) at 61%, 73% and 62% of the predicted values respectively. Echocardiography revealed tricuspid regurgitation +1 and mild enlargement of the right atrium.

Acute renal and respiratory failure was confirmed as the cause of death in a patient (pt VI-Table 2) with a history of chronic obstructive pulmonary disease, medically controlled hypothyroidism and aortocoronary bypass 8 years ago. Spirometry revealed mild abnormalities (FEV_1_: 68% and FVC: 72% of the predicted values) while assessment of the arterial blood gases did not disclose any severe hypoxemia or hypercapnia. He had not experienced any symptoms of coronary ischemia and he met a 5-MET level before surgery.

Paralytic ileus was the cause of death in a patient with insignificant history (pt VII-Table 2) while another patient with a free medical history died from septic shock complicating a case of pneumonia (pt VIII-Table 2).

*Intraoperative complications *occurred in 22 patients (44%) with mild hypotension being the most common. Intraoperative, a temporary fall in blood arterial pressure (BAP) was common in almost all patients. Red blood cells had to be transfused to 32 out of 50 patients (64%) intraoperative due to excessive blood loss. None patient died in the operating table or during the hospitalization period. None patient had to be transported to the intensive care unit and also none presented with major postoperative complications during the early postoperative period.

Four of the diseased patients had hemodynamic imbalance intraoperative (50%) and 7 out of 8 received blood intraoperative (87,5%). The incidence of intraoperative complications in this group of patients was similar to that of our original group of patients (50% vs. 44%, p = 0.037, and 64% vs 87,5% p < 0,05).

## Discussion

Elderly patients suffering from cardiovascular disease undergoing non-cardiac surgery are plagued with high cardiovascular morbidity representing a challenge for the anesthesiologic and surgical team. For patients over 70, there is a consistent 3-fold increase in the mortality rate and according to Goldman, age is one of nine independent predictor factors of morbidity and death [7,8]. In our study, six of the eight patients who died had a history of cardiovascular disease. Five of the 8 patients died because of cardiovascular disease; ischemic heart disease and MI were the causes of death in four patients and heart failure in one.

Autopsies of elderly people have revealed ischemia and heart failure as the causes of death for the majority of patients [9]. As Zeldin points out, more than 80% of deaths attributed to cadiovascular disease occur in people over the age of 65 [10]. In our study, the average clinical status, which was evaluated by the MET-levels (all these 6 patients met a 5–6 MET-level), and their tests results (ECG, echocardiography) did not predict an ominous outcome. Increased perioperative and long-term risk of cardiac events has been associated with the inability to function above 4 MET [2]. Still, test results very often do not disclose the severity of the underlying cardiovascular disease [11].

The pre-assessment, clinical findings and echocardiography failed to reveal the severity of aortic valve stenosis in a patient with a history of this disease. That patient died within a month after surgery from MI. Aortic valve stenosis has been recognized as a major independent risk factor for perioperative cardiac events in patients undergoing non-cardiac surgery. Patients with aortic valve stenosis have a 5-fold increased risk of perioperative complications in comparison to patients without aortic valve stenosis [12].

One patient with coexisting chronic obstructive pulmonary disease died because of respiratory failure although he had only mild abnormalities in the pulmonary function tests (PFTs). There is evidence that PFTs are not sensitive enough to predict the risk of postoperative pulmonary complications. PFTs have low positive predictive value and should be combined with other assessments such as ASA physical status and the Shapiro classification [13].

The patients who died had significant greater blood transfusion requirements compared with the initial group of patients enrolled in the study (87,5% vs. 64%, p = 0,03). There is evidence that fluid and blood transfusion might predict myocardial infarction, sepsis and influence mortality [14]. The threshold for blood transfusion was less than 10 mg.dL^-1 ^for patients with cardiac disease and 8 mg.dl^-1 ^for the rest, although some authors insist in blood transfusion when haemoglobin falls below 8 mg/dL or alternatively 7 g.dL^-1 ^[15]. Maintaining very low thresholds for transfusion may not be appropriate in the elderly. Even moderate anaemia is poorly tolerated by patients with cardiovascular disease in the perioperative phase. We did not follow a strict transfusion threshold because coronary ischemia is frequently silent in the elderly, in both the pre- and postoperative period. In addition, anaemia in the elderly, even when haemoglobin levels are above 12 g.dL^-1^, is a strong predictor of functional decline and poor prognosis.

Our findings on mortality are broadly comparable with those in other studies of mortality after fractured neck of femur or hip replacement in defined populations [5,16-20]. In a prospective study of 2448 patients over 60 years of age suffering from hip fracture, Roche et al reported mortality rates of 9.6% at 30 days and 33% at one year [16]. Lie at al, also found increased mortality rates in patients over 70 years after hip replacement [5].

To reduce mortality, attention must focus on optimising health status preoperatively, preventing postoperative complications and when these complications develop providing optimal specialist care. However, it seems that in spite of a detailed preoperative evaluation still many conditions remain unidentified even in the operating room. In 27–31% of autopsies of patients over 65 years of age, the presence of a potentially treatable contributory factor to death that was undiagnosed during life was identified [21]. Approximately 80–90% of patients with obstructive sleep apnea are undiagnosed [22]. Even laboratory screening tests have several shortcomings: they frequently fail to uncover pathological conditions; they detect abnormalities the discovery of which does not necessarily improve patients care outcome; and they are inefficient in finding asymptomatic diseases [11].

It is difficult to determine the gold standard method for perioperative management of these patients. For example Gold Directed Haemodynamic Therapy guided by oxygen extraction O_2_ER is very helpful but it could not be performed in our study since it would require central venous monitoring for all patients [23]. Also B-type natriuretic peptide (BNP) is a well known marker of left ventricular dysfunction and heart failure, it provides prognostic information beyond and above left ventricular ejection fraction and is an important and independent prognostic biomarker for both short- and long-term mortality in patients with acute coronary syndromes or chest pain. However, its prognostic value is minimized in asymptomatic patients as in our study [24,25]. Anesthesiologists and all physicians who deal with the peri- and post-operative management of these patients need to be vigilant for any complications that might arise.

Certain limitations of this study are its small population and its short follow up. As we had only 50 patients in our series, a larger population is necessary to confirm our results and explain the cause of misdiagnosis or delayed right diagnosis. The study is retrospective so there could not be obtained a detailed history of patients presented in the ER. Future prospective studies are needed to evaluate the utility of each screening test in the ER for patients suspicious for AAD. There was no control group, as it would not be ethical not to perform screening tests in a cohort of patients. Certainly the findings of our study can not be generalized, but are indicative of how commonly happens misdiagnosis in AAD.

## Conclusion

Postoperative mortality and morbidity are related to age, however the severity and the increased frequency of the underlying medical problems appear to be significant risk factors as well. Despite the detailed preoperative evaluation, and the intensive intraoperative and early postoperative anaesthetic care, most patients' deaths are directly correlated to their previous medical history.

## Competing interests

The authors declare that they have no competing interests.

## Authors' contributions

AI participated in the design of the study and drafted the manuscript. AT was a major ****contributor in writing the manuscript. GG participated in the design of the study. PS and KN edited and revised the final manuscript. All authors have read and approved the final manuscript.
